# A case report of *SPG11* mutations in a Chinese ARHSP-TCC family

**DOI:** 10.1186/s12883-016-0604-5

**Published:** 2016-06-03

**Authors:** Linwei Zhang, Karen N. McFarland, Jinsong Jiao, Yujuan Jiao

**Affiliations:** Department of Neurology, China-Japan Friendship Hospital, 2 Yinghua Dongjie, Hepingli, 100029 Beijing, China; McKnight Brain Institute and the Department of Neurology, College of Medicine, University of Florida, Gainesville, FL 32610 United States of America

**Keywords:** Autosomal recessive hereditary spastic paraplegia with thin corpus callosum (ARHSP-TCC), *SPG11*, Gene mutation, Heterozygous mutations

## Abstract

**Background:**

Autosomal recessive hereditary spastic paraplegia (ARHSP) with thin corpus callosum (TCC) is a complicated form of hereditary spastic paraplegia, characterized by progressive spastic paraplegia, weakness of the lower extremities and is usually accompanied by mental retardation. Mutations in the *Spastic Paraplegia gene 11* (*SPG11*) account for a large proportion of ARHSP-TCC cases worldwide.

**Case presentation:**

We describe a Chinese family with ARHSP-TCC. Two daughters of this family presented with a spastic gait and cognitive impairment. Brain imaging of the index patient revealed a thin corpus callosum. We performed detailed physical and auxiliary examinations and were able to exclude acquired causes of spastic paraplegia. To determine the causative mutation, we took a candidate gene approach and screened the coding sequence and some flanking intronic sequence of *SPG11* by direct Sanger sequencing. We identified two novel compound heterozygous mutations in *SPG11* in affected individuals (c.1551_1552delTT, p.Cys518SerfsTer39 and c.5867-1G > T (IVS30-1G > T), p.Thr1956ArgfsTer15). Bioinformatic analysis predicts that these mutations would lead to a loss of protein function due to the truncation of the *SPG11* protein.

**Conclusions:**

The results of this case report indicate a broader approach to include screening for *SPG11* mutations in ARHSP-TCC patients. Our findings enrich the phenotypic spectrum of *SPG11* mutations.

**Electronic supplementary material:**

The online version of this article (doi:10.1186/s12883-016-0604-5) contains supplementary material, which is available to authorized users.

## Background

Hereditary spastic paraplegia (HSP) is a kind of neurodegenerative disease characterized by progressive weakness and spasticity of the lower limbs. Depending on the mode of inheritance, it can be classified as autosomal dominant, autosomal recessive or X-linked. In complicated forms, additional neurological signs, such as ataxia, mental retardation, epilepsy, peripheral neuropathies, are present [1]. HSP with Thin Corpus Callosum (HSP-TCC) is one of the most common complicated forms of autosomal recessive hereditary spastic paraplegia (ARHSP). ARHSP-TCC is most commonly caused by mutations in the *spastic paraplegia 11* (*SPG11*) gene [2]. *SPG11* maps to chromosome 15q21, encodes a 2443 amino acid protein, SPATACSIN, and is widely expressed in the nervous system, particularly in the neurons of the cerebellum and cerebral cortex [3]. Here, we report a Chinese HSP-TCC non-consanguineous family whose affected family members possess two compound heterozygous mutations in *SPG11*. These results implicate a need for wider screening of *SPG11* mutations in ARHSP-TCC patients.

## Case presentation

**Fig. 1:**
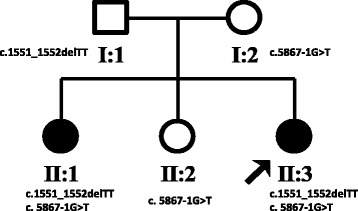
Family pedigree of non-conanguineous parents indicating segregation of *SPG11* mutations. *Squares*, male; *circles*, female; *filled circle*, affected female; *arrow*, proband

**Fig. 2:**
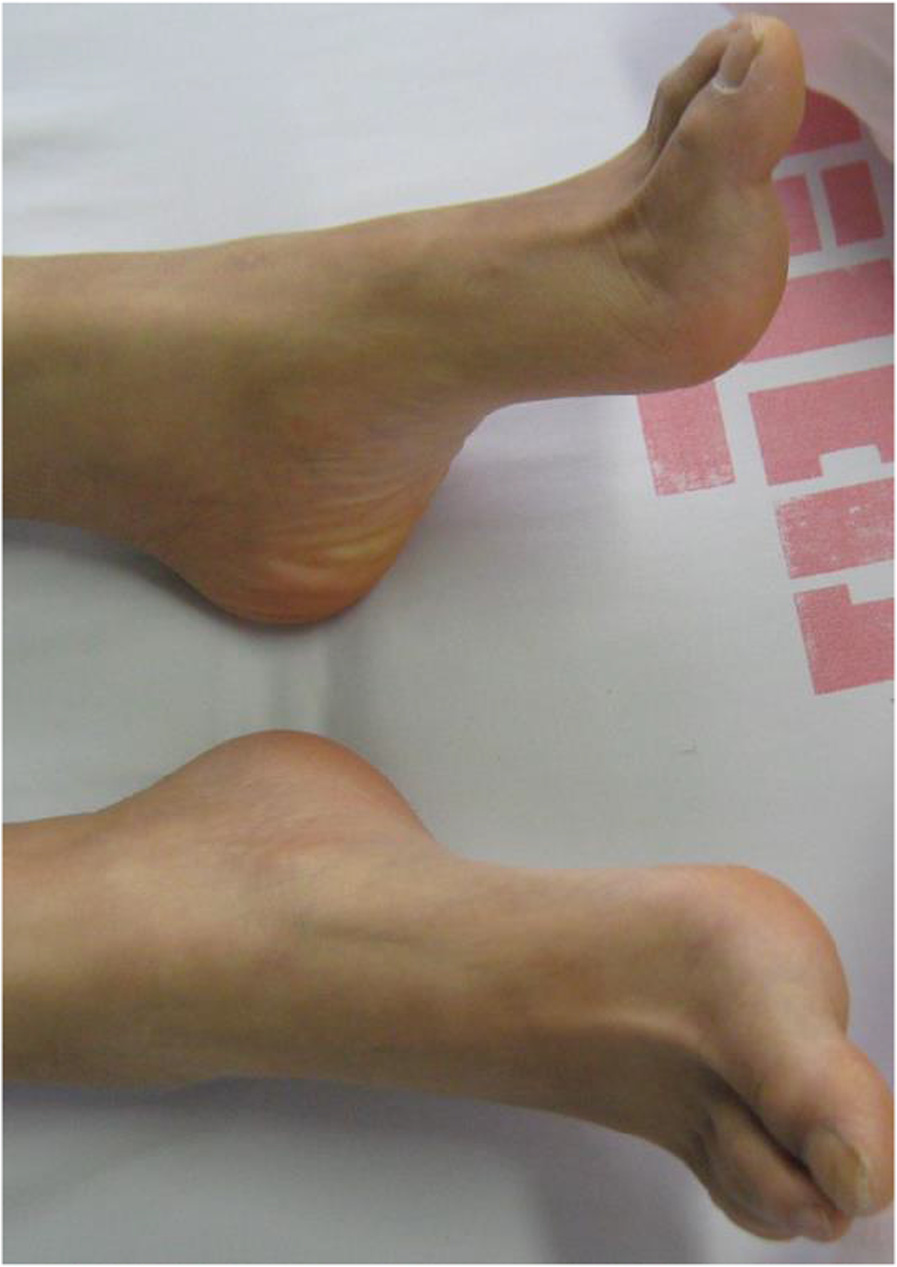
Presentation of pes cavus in the index patient (II:3)

**Fig. 3:**
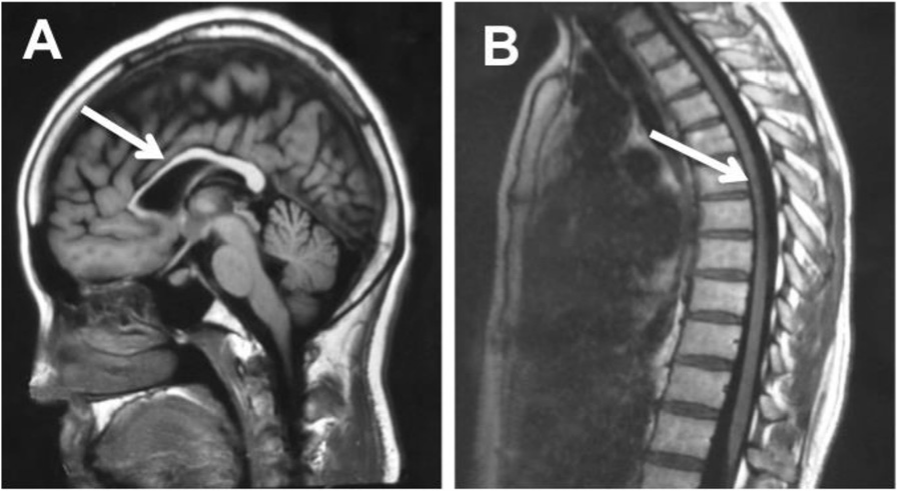
Sagittal brain and thoracic spinal MRI image in index patient (II:3). **a**: Brain MRI shows thinning of corpus callosum, emphasized in the anterior part with a “beaked” shape (*arrow*). Ther is no obvious periventricular or deep cerebellar white matter lesions and no obvious cerebral or cerebellar atrophy. **b**: Throracic spinal MRI shows thinning of the thoracic spinal cord with volume loss but no obvious signal abnormalities in the cord

Index Patient (II:3) is a 26-year old female with healthy non-consanguineous parents (Fig. 1). Beginning at the age of 20, she developed weakness in the lower limbs, accompanied by spasticity which was not alleviated by treatment with baclofen. She is cognitively impaired, advancing in her education only until middle school. MMSE and MoCA scores were 23/30 and 15/30, respectively (impaired in execution, calculation and delayed memory). Upon examination, she had mild dysarthria, bilateral patellar clonus, positive Babinski’s sign, pes cavus (Fig. 2) and a scissors gait. Upper extremities were apparently normal. Blood and CSF testing showed no marked abnormalities. EMG revealed neurogenic, mild axonal sensory-motor neuropathy, especially in lower extremities. Brain MRI revealed extreme thinning of corpus callosum, particularly in the anterior region (Fig. 3a). Thoracic MRI showed remarkable atrophy of thoracic spinal cord (Fig. 3b).

Her sister (II-1) is a 33-year old female with a similar, but more severe clinical phenotype with an 18 year history of disease. She is bedridden and severely handicapped due to the disturbance in her gait. She has severe cognitive impairment and did not progress past an elementary school education.

**Fig. 4:**
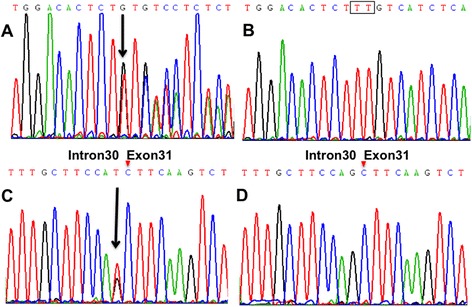
Sanger sequencing of exon 7 (c.1551_1552delTT) containing a TT deletion in *SPG11* in an affected individual (**a**) and unaffected individual (**b**). Sanger sequencing of a splice site mutation (c.5867-1G > T (IVS30-1G > T)) in intron 30 of *SPG11* in an affected individual (**c**) and control, unaffected (**d**)

Genomic DNA was extracted from peripheral blood samples according to standard procedures. *SPG11* was screened for mutations by PCR amplification using previously described primer pairs [4] followed by Sanger sequencing of the amplicons (Additional file 1). In the two affected individuals, compound heterozygous mutations were identified in the *SPG11* gene: c.1551_1552delTT, p.Cys518SerfsTer39 in exon 7; and c.5867-1G > T (IVS30-1G > T), p.Thr1956ArgfsTer15, a splice site mutation in intron 30 (showed in Fig. 4). Segregation of the genetic variants was validated in the other family members: the father (I:1) carried c.1551_1552delTT in the heterozygous state, while the mother (I:2) and the middle sister (II:2) carried c.5867-1G > T (IVS30-1G > T) in the heterozygous state (see Fig. 1). Both parents and the middle sister showed no symptom of spasticity or cognitive deterioration. The c.1551_1552delTT is novel but is similar to two previously described, nearby deletion mutations at c.1550_1551delTT (rs312262730) and c.1549_1550delCT (rs312262730), both of which are associated with *SPG11* phenotypes. The second mutation, c.5867-1G > T (IVS30-1G > T), is also novel. Neither of these mutations was detected in 100 unrelated healthy Chinese control individuals or in the 1000 Genomes database.

**Fig. 5:**
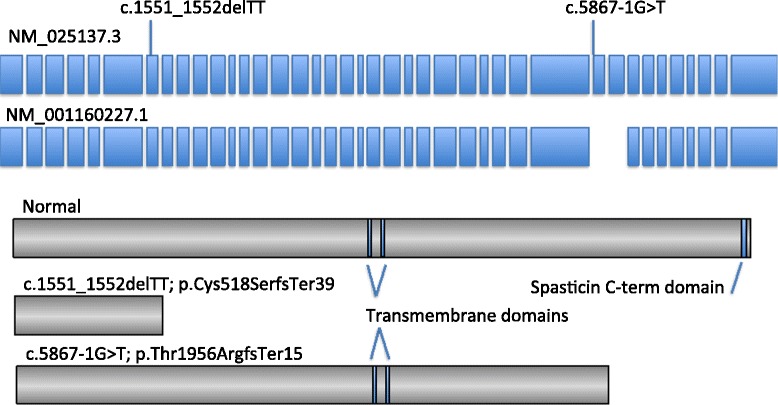
Bioinformatic prediction of the SPG11 protein structure in the presence of the found mutations. *Boxes in blue* indicate the exons of the SPG11 major transcripts (NM_025137 and NM_001160227). The location of the mutations described in this study are located in exon 7 (c.1551_1552delTT) and upstream of exon 31 (c.5867-1G > T (IVS30-1G > T)). *Boxes in grey* indicate the predicated protein truncations (*middle and lower*) as compared with a full-length wild-type structure (*upper*)

Bioinformatic analysis of these mutations is predicted to prematurely truncate the protein product. The c.1551_1552delTT mutations causes a frameshift at amino acid reside 518 resulting in a premature stop codon (p.Cys518SerfsTer39). Such a mutation would result in a truncated protein but more likely would result in reduced transcript levels due to non-sense mediated decay. Similar mutations have been described in other cases of *SPG11* [5]. The second mutation alters the consensus AG sequence at the 3′ acceptor site of intron 30 (c.5867-1G > T (IVS30-1G > T)). Splice site predictions using the mutated sequence were performed using Splice Site Prediction by Neural Network (http://www.fruitfly.org/seq_tools/splice.html) and NetGene2 (http://www.cbs.dtu.dk/services/NetGene2/). Both analysis programs predict a complete abolishment of splicing using the mutated 3′ acceptor site. Thus, we predict exclusion of exon 31 from the transcript resulting in a frameshift and premature stop codon shortly downstream (p.Tyr1956ArgfsTer15). Similar to the above mutation, this would likely result in a truncated protein or alternatively may result in reduced transcript levels due to nonsense mediated decay, suggesting a possible loss of function mechanism (Fig. 5).

## Conclusions

We identified novel compound heterozygous mutations in *SPG11* in a Chinese HSP-TCC family (c.1551_1552delTT and c.5867-1G > T (IVS30-1G > T)). Our patients presented with typical clinical symptoms of ARHSP-TCC which included spastic paraplegia, cognitive impairment, peripheral neuropathy and distal musle atrophy. Thinning of thoracic spinal cord in our index patient is likely the result of degeneration of long corticospinal tracts and is usually present in nearly all subtypes of the HSP, mostly at later disease stages [6, 7].

Mutations in *SPG11* are found in the majority of reported complex ARHSP-TCC cases with TCC being the single best indicator for *SPG11* [2]. Another Chinese family with ARHSP-TCC was reported previously, the index patient presented with prominent intellectual disability rather than spasticity had different compound heterozygous mutations of *SPG11* [8]. For patients presenting with HSP-TCC, *SPG11* should be screened initially once infectious causes are eliminated. Other SPG loci including SPG15, SPG35 and SPG48 should be considered if no mutations in *SPG11* are discovered [9]. Interestingly, mutations in *SPG11* can also cause other disorders, such as juvenile amyotrophic lateral sclerosis (ALS5) [10], juvenile Parkinsonism [11], and autosomal recessive axonal Charcot-Marie-Tooth disease [12]. Thus, *SPG11* mutations have an wide phenotypic spectrum suggesting that additional care should be taken when examining HSP patients. In summary, we demonstrate the importance of screening the *SPG11* gene in the ARHSP-TCC patients and have provided additional clinical phenotypes resulting from mutations in the *SPG11* gene.

## Abbreviations

AR, autosomal recessive; CSF, cerebrospinal fluid; EMG, electromyography; HSP, hereditary spastic paraplegia; MMSE, mini-mental state examination; MoCA, Montreal cognitive assessment; MRI, magnetic resonance imaging; SPG, spastic paraplegia gene; TCC, thin corpus callosum.

## Additional files

Additional file 1: Primer sequences and reaction conditions used for the 40 exons of *SPG11* gene. (DOCX 300 kb) Additional file 2: Splice Site Prediction by Neural Network. (PPTX 77 kb)
