# Supplementary material for: A case report of SPG11 mutations in a Chinese ARHSP-TCC family
Source: BMC Neurol. 2016 Jun 3;16:87. doi: 10.1186/s12883-016-0604-5 (PMC4891852; doi:10.1186/s12883-016-0604-5)
Supplement: Additional file 1: — Primer sequences and reaction conditions used for the 40 exons of SPG11 gene. (DOCX 300 kb) [file 12883_2016_604_MOESM1_ESM.docx]

**KIAA1840-E1**

1. KIAA1840-E1-F：cca cag gaa acg aat gga at

2. KIAA1840-E1-R：ggt tct gtg agg aaa cca cg

3.length：572bp

4.system：

|  | 1× |
| --- | --- |
| ddH_2_O | 4.8 |
| 2mM dNTP | 2.5 |
| 5uM KIAA1840-E1-F | 1.5 |
| 5uM KIAA1840-E1-R | 1.5 |
| 2×GCbufferI | 12.5 |
| LA-Taq | 0.2 |
| template | 2 |

5. Procedure：

94℃5min→94℃30S→62℃30S→72℃45S→72℃10min→4℃

×35

**KIAA1840-E2**

1. KIAA1840-E2-F：ctg agc ccc aca ttt ttg tt

2. KIAA1840-E2-R：caa gtg ctc aat agc ccc at

3.length：569bp

4.system：

|  | 1× |
| --- | --- |
| ddH_2_O | 14.8 |
| 2mM dNTP | 2.5 |
| 5uM KIAA1840-E2-F | 1.5 |
| 5uM KIAA1840-E2-R | 1.5 |
| 10×buffer | 2.5 |
| r-Taq | 0.2 |
| template | 2 |

5. Procedure：

94℃5min→94℃30S→64℃30S→72℃45S→72℃10min→4℃

×35

**KIAA1840-E3**

1.KIAA1840-E3-F：cag gga cat tgt agg cca tc

2.KIAA1840-E3-R：tcc cag ctc cca aaa cta aa

3.length：525bp

4.system：

|  | 1× |
| --- | --- |
| ddH_2_O | 14.8 |
| 2mM dNTP | 2.5 |
| 5uM KIAA1840-E3-F | 1.5 |
| 5uM KIAA1840-E3-R | 1.5 |
| 10×buffer | 2.5 |
| r-Taq | 0.2 |
| template | 2 |

5. Procedure：

94℃5min→94℃30S→62℃30S→72℃45S→72℃10min→4℃

×35

**KIAA1840-E4**

1.KIAA1840-E4-F：cag gtt ctt tat tgt ggc atc a

2.KIAA1840-E4-R：cga gga tat ttt taa cct ctt atc a

3.length：462 bp

4.system：

|  | 1× |
| --- | --- |
| ddH_2_O | 14.8 |
| 2mM dNTP | 2.5 |
| 5uM KIAA1840-E4-F | 1.5 |
| 5uM KIAA1840-E4-R | 1.5 |
| 10×buffer | 2.5 |
| r-Taq | 0.2 |
| template | 2 |

5. Procedure：

94℃5min→94℃30S→56℃30S→72℃45S→72℃10min→4℃

×35

**KIAA1840-E5**

1.KIAA1840-E5-F：gct aac tgc cct taa tag agt aaa a

2.KIAA1840-E5-R：aaa ggg tac agc gtc agc at

3.length：406bp

4.system：

|  | 1× |
| --- | --- |
| ddH_2_O | 14.8 |
| 2mM dNTP | 2.5 |
| 5uM KIAA1840-E5-F | 1.5 |
| 5uM KIAA1840-E5-R | 1.5 |
| 10×buffer | 2.5 |
| r-Taq | 0.2 |
| template | 2 |

5. Procedure：

94℃5min→94℃30S→64℃30S→72℃45S→94℃30S→49℃30S→72℃45S→72℃10min→4℃

×15（-1℃/cycle）

×20

**KIAA1840-E6**

1.KIAA1840-E6-F：gaa cat ctt tgc cct ggt tt

2.KIAA1840-E6-R：cag gca ctg agg cag aag ta

3.length：612bp

4. Procedure：

|  | 1× |
| --- | --- |
| ddH_2_O | 14.8 |
| 2mM dNTP | 2.5 |
| 5uM KIAA1840-E6-F | 1.5 |
| 5uM KIAA1840-E6-R | 1.5 |
| 10×buffer | 2.5 |
| r-Taq | 0.2 |
| template | 2 |

5. Procedure：

94℃5min→94℃30S→64℃30S→72℃45S→72℃10min→4℃

×35

**KIAA1840-E7**

1.KIAA1840-E7-F：aaa aat caa ttc cta aat cat aat cc

2.KIAA1840-E7-R：tct ttt aaa gcc aaa aag ggt aaa

3.length：412bp

4.system：

|  | 1× |
| --- | --- |
| ddH_2_O | 14.8 |
| 2mM dNTP | 2.5 |
| 5uM KIAA1840-E7-F | 1.5 |
| 5uM KIAA1840-E7-R | 1.5 |
| 10×buffer | 2.5 |
| r-Taq | 0.2 |
| template | 2 |

5. Procedure：

94℃5min→94℃30S→56℃30S→72℃45S→72℃10min→4℃

×35

**KIAA1840-E8**

1.KIAA1840-E8-F：ctt gcc cca gat tgc ata at

2.KIAA1840-E8-R：tcc aaa aag tac gta aaa tcc ca

3.length：449bp

4.system：

|  | 1× |
| --- | --- |
| ddH_2_O | 14.8 |
| 2mM dNTP | 2.5 |
| 5uM KIAA1840-E8-F | 1.5 |
| 5uM KIAA1840-E8-R | 1.5 |
| 10×buffer | 2.5 |
| r-Taq | 0.2 |
| template | 2 |

5. Procedure：

94℃5min→94℃30S→64℃30S→72℃45S→72℃10min→4℃

×35

**KIAA1840-E9**

1.KIAA1840-E9-F：cag caa aag ggt aat agc agtg

2.KIAA1840-E9-R：ccc aaa tgt agt aaa tgg cg

3.length：499bp

4.system：

|  | 1× |
| --- | --- |
| ddH_2_O | 14.8 |
| 2mM dNTP | 2.5 |
| 5uM KIAA1840-E9-F | 1.5 |
| 5uM KIAA1840-E9-R | 1.5 |
| 10×buffer | 2.5 |
| r-Taq | 0.2 |
| template | 2 |

5. Procedure：

94℃5min→94℃30S→63℃30S→72℃45S→72℃10min→4℃

×35

**KIAA1840-E10**

1.KIAA1840-E10-F：ccc agg act aat cat gaa gga

2.KIAA1840-E10-R：atc ccc aaa ccg ata aaa cc

3.length：526bp

4.system：

|  | 1× |
| --- | --- |
| ddH_2_O | 14.8 |
| 2mM dNTP | 2.5 |
| 5uM KIAA1840-E10-F | 1.5 |
| 5uM KIAA1840-E10-R | 1.5 |
| 10×buffer | 2.5 |
| r-Taq | 0.2 |
| template | 2 |

5. Procedure：

94℃5min→94℃30S→60℃30S→72℃45S→72℃10min→4℃

×35

**KIAA1840-E11**

1.KIAA1840-E11-F：cgg tgt gtc ttc cac tag ctc

2.KIAA1840-E11-R：acc cag cca ttc tca gtg tt

3.length：539bp

4.system：

|  | 1× |
| --- | --- |
| ddH_2_O | 14.8 |
| 2mM dNTP | 1.5 |
| 5uM KIAA1840-E11-F | 2.5 |
| 5uM KIAA1840-E11-R | 1.5 |
| 10×buffer | 2.5 |
| r-Taq | 0.2 |
| template | 2 |

5. Procedure：

94℃5min→94℃30S→64℃30S→72℃45S→72℃10min→4℃

×35

**KIAA1840-E12**

1.KIAA1840-E12-F：ttt gaa aga gca gaa agc tat gg

2.KIAA1840-E12-R：tga agg ggt tgt cac act ttt

3.length：350bp

4.system：

|  | 1× |
| --- | --- |
| ddH_2_O | 14.8 |
| 2mM dNTP | 2.5 |
| 5uM KIAA1840-E12-F | 1.5 |
| 5uM KIAA1840-E12-R | 1.5 |
| 10×buffer | 2.5 |
| r-Taq | 0.2 |
| template | 2 |

5. Procedure：

94℃5min→94℃30S→59℃30S→72℃45S→72℃10min→4℃

×35

**KIAA1840-E13**

1.KIAA1840-E13-F：ttg tgg caa aag aaa att tgt g

2.KIAA1840-E13-R：gag aat gca ggc tca gtt cc

3.length：433bp

4.system：

|  | 1× |
| --- | --- |
| ddH_2_O | 14.8 |
| 2mM dNTP | 2.5 |
| 5uM KIAA1840-E13-F | 1.5 |
| 5uM KIAA1840-E13-R | 1.5 |
| 10×buffer | 2.5 |
| r-Taq | 0.2 |
| template | 2 |

5. Procedure：

94℃5min→94℃30S→59℃30S→72℃45S→72℃10min→4℃

×35

**KIAA1840-E14**

1.KIAA1840-E14-F：atg tgg aac tga gcc tgc at

2.KIAA1840-E14-R：cga ctt gca ttt taa aga acc tg

3.length：512bp

4.system：

|  | 1× |
| --- | --- |
| ddH_2_O | 14.8 |
| 2mM dNTP | 2.5 |
| 5uM KIAA1840-E14-F | 1.5 |
| 5uM KIAA1840-E14-R | 1.5 |
| 10×buffer | 2.5 |
| r-Taq | 0.2 |
| template | 2 |

5.procedure：

94℃5min→94℃30S→59℃30S→72℃45S→72℃10min→4℃

×35

**KIAA1840-E15**

1.KIAA1840-E15-F：cac agc gag atc ctg tct ca

2.KIAA1840-E15-R：cct cac tgt aag atg atg ccc

3.length：542bp

4.system：

|  | 1× |
| --- | --- |
| ddH_2_O | 14.8 |
| 2mM dNTP | 2.5 |
| 5uM KIAA1840-E15-F | 1.5 |
| 5uM KIAA1840-E15-R | 1.5 |
| 10×buffer | 2.5 |
| r-Taq | 0.2 |
| template | 2 |

5.procedure：

94℃5min→94℃30S→65℃30S→72℃45S→94℃30S→50℃30S→72℃45S→72℃10min→4℃

×20

×15（-1℃/cycle）

**KIAA1840-E16**

1.KIAA1840-E16-F：cct tta aat act aca gtg gtg cag a

2.KIAA1840-E16-R：cca act gtt gag atg gag aaa a

3.length：551bp

4.system：

|  | 1× |
| --- | --- |
| ddH_2_O | 14.8 |
| 2mM dNTP | 2.5 |
| 5uM KIAA1840-E16-F | 1.5 |
| 5uM KIAA1840-E16-R | 1.5 |
| 10×buffer | 2.5 |
| r-Taq | 0.2 |
| template | 2 |

5. Procedure：

94℃5min→94℃30S→54℃30S→72℃45S→72℃10min→4℃

×35

**KIAA1840-E17**

1.KIAA1840-E17-F：ttg ttt cca gat cat gaa gaa tat g

2.KIAA1840-E17-R：tca gat agc tga cca cag cc

3.length：400bp

4.system：

|  | 1× |
| --- | --- |
| ddH_2_O | 14.8 |
| 2mM dNTP | 2.5 |
| 5uM KIAA1840-E17-F | 1.5 |
| 5uM KIAA1840-E17-R | 1.5 |
| 10×buffer | 2.5 |
| r-Taq | 0.2 |
| template | 2 |

5. Procedure：

94℃5min→94℃30S→59℃30S→72℃45S→72℃10min→4℃

×35

**KIAA1840-E18**

1.KIAA1840-E18-F：tcc ctc tta agg aga aaa aca ctg

2.KIAA1840-E18-R：acc ggg ccg aga tat aaa at

3.length：354bp

4.system：

|  | 1× |
| --- | --- |
| ddH_2_O | 14.8 |
| 2mM dNTP | 2.5 |
| 5uM KIAA1840-E18-F | 1.5 |
| 5uM KIAA1840-E18-R | 1.5 |
| 10×buffer | 2.5 |
| r-Taq | 0.2 |
| template | 2 |

5. Procedure：

94℃5min→94℃30S→58℃30S→72℃45S→72℃10min→4℃

×35

**KIAA1840-E19**

1.KIAA1840-E19-F：gct agt ttg tct tag aac cag aac a

2.KIAA1840-E19-R：ttt tgg gtt gtc tca cta tca ca

3.length：502bp

4.system：

|  | 1× |
| --- | --- |
| ddH_2_O | 14.8 |
| 2mM dNTP | 2.5 |
| 5uM KIAA1840-E19-F | 1.5 |
| 5uM KIAA1840-E19-R | 1.5 |
| 10×buffer | 2.5 |
| r-Taq | 0.2 |
| template | 2 |

5. Procedure：

94℃5min→94℃30S→63℃30S→72℃45S→72℃10min→4℃

×35

**KIAA1840-E20**

1.KIAA1840-E20-F：aag gaa cat agc cag ttc tgt ttt

2.KIAA1840-E20-R：tgc gaa cta ttt ttc ctt tgg

3.length：421bp

4.system：

|  | 1× |
| --- | --- |
| ddH_2_O | 14.8 |
| 2mM dNTP | 2.5 |
| 5uM KIAA1840-E20-F | 1.5 |
| 5uM KIAA1840-E20-R | 1.5 |
| 10×buffer | 2.5 |
| r-Taq | 0.2 |
| template | 2 |

5. Procedure：

94℃5min→94℃30S→58℃30S→72℃45S→72℃10min→4℃

×35

**KIAA1840-E21**

1.KIAA1840-E21-F：ggc tga agc atc att tct tac c

2.KIAA1840-E21-R：gtg aac cac tgt acc cag aca a

3.length：452bp

4.system：

|  | 1× |
| --- | --- |
| ddH_2_O | 14.8 |
| 2mM dNTP | 2.5 |
| 5uM KIAA1840-E21-F | 1.5 |
| 5uM KIAA1840-E21-R | 1.5 |
| 10×buffer | 2.5 |
| r-Taq | 0.2 |
| template | 2 |

5. Procedure：

94℃5min→94℃30S→64℃30S→72℃45S→72℃10min→4℃

×35

**KIAA1840-E22**

1.KIAA1840-E22-F：agt cag ctt aag gga agc gg

2.KIAA1840-E22-R：gaa gat aac cat ttt ctc ccc a

3.length：519bp

4.system：

|  | 1× |
| --- | --- |
| ddH_2_O | 14.8 |
| 2mM dNTP | 2.5 |
| 5uM KIAA1840-E22-F | 1.5 |
| 5uM KIAA1840-E22-R | 1.5 |
| 10×buffer | 2.5 |
| r-Taq | 0.2 |
| template | 2 |

5. Procedure：

94℃5min→94℃30S→64℃30S→72℃45S→94℃30S→49℃30S→72℃45S→72℃10min→4℃

×20

×15（-1℃/cycle）

**KIAA1840-E23**

1.KIAA1840-E23-F：ttg tga gtg ttt ggg gag aa

2.KIAA1840-E23-R：ggg gat tta gtg aaa aca cca

3.length：405bp

4.system：

|  | 1× |
| --- | --- |
| ddH_2_O | 14.8 |
| 2mM dNTP | 2.5 |
| 5uM KIAA1840-E23-F | 1.5 |
| 5uM KIAA1840-E23-R | 1.5 |
| 10×buffer | 2.5 |
| r-Taq | 0.2 |
| template | 2 |

5. Procedure：

94℃5min→94℃30S→58℃30S→72℃45S→72℃10min→4℃

×35

**KIAA1840-E24**

1.KIAA1840-E24-F：ttt gtt gga gaa tac act gtg ctt

2.KIAA1840-E24-R：cat gtc tac aca aca gaa aga atg c

3.length：370bp

4.system：

|  | 1× |
| --- | --- |
| ddH_2_O | 14.8 |
| 2mM dNTP | 2.5 |
| 5uM KIAA1840-E24-F | 1.5 |
| 5uM KIAA1840-E24-R | 1.5 |
| 10×buffer | 2.5 |
| r-Taq | 0.2 |
| template | 2 |

5. Procedure：

94℃5min→94℃30S→63℃30S→72℃45S→72℃10min→4℃

×35

**KIAA1840-E25**

1.KIAA1840-E25-F：aaa agg cac cat aca gct ttg

2.KIAA1840-E25-R：gga aac aca tgc tgg aac ct

3.length：552bp

4. Procedure：

|  | 1× |
| --- | --- |
| ddH_2_O | 14.8 |
| 2mM dNTP | 2.5 |
| 5uM KIAA1840-E25-F | 1.5 |
| 5uM KIAA1840-E25-R | 1.5 |
| 10×buffer | 2.5 |
| r-Taq | 0.2 |
| template | 2 |

5. Procedure：

94℃5min→94℃30S→63℃30S→72℃45S→72℃10min→4℃

×35

**KIAA1840-E26**

1.KIAA1840-E26-F：ctt ctg tct gct tct tgg tct t

2.KIAA1840-E26-R：tat cat cat tat ctg ttg ttg g

3.length：542bp

4.system：

|  | 1× |
| --- | --- |
| ddH_2_O | 14.8 |
| 2mM dNTP | 2.5 |
| 5uM KIAA1840-E26-F | 1.5 |
| 5uM KIAA1840-E26-R | 1.5 |
| 10×buffer | 2.5 |
| r-Taq | 0.2 |
| template | 2 |

5. Procedure：

94℃5min→94℃30S→62℃30S→72℃45S→72℃10min→4℃

×35

**KIAA1840-E27**

1.KIAA1840-E27-F：tta ggt gat ccc act ggc tc

2.KIAA1840-E27-R：ccc agg agt tca agg ctg ta

3.length：423bp

4.system：

|  | 1× |
| --- | --- |
| ddH_2_O | 4.8 |
| 2mM dNTP | 2.5 |
| 5uM - KIAA1840-E27-F | 1.5 |
| 5uM - KIAA1840-E27-R | 1.5 |
| 2×GC buffer Ⅰ | 12.5 |
| LA-Taq | 0.2 |
| template | 2 |

5. Procedure：

94℃5min→94℃30S→66℃30S→72℃45S→94℃30S→51℃30S→72℃45S→72℃10min→4℃

×20

×15（-1℃/cycle）

**KIAA1840-E28**

1.KIAA1840-E28-F：ctg agg agg gct tgt ttt tg

2.KIAA1840-E28-R：tct gta act tgt tta ctc cca gtt g

3.length：501bp

4.system：

|  | 1× |
| --- | --- |
| ddH_2_O | 14.8 |
| 2mM dNTP | 2.5 |
| 5uM KIAA1840-E28-F | 1.5 |
| 5uM KIAA1840-E28-R | 1.5 |
| 10×buffer | 2.5 |
| r-Taq | 0.2 |
| template | 2 |

5. Procedure：

94℃5min→94℃30S→61℃30S→72℃45S→72℃10min→4℃

×35

**KIAA1840-E29**

1.KIAA1840-E29-F：acg tat att ttc ccc ctg ata tgt

2.KIAA1840-E29-R：agc aag acc ccg tat cta aaa a

3.length：579bp

4.system：

|  | 1× |
| --- | --- |
| ddH_2_O | 14.8 |
| 2mM dNTP | 2.5 |
| 5uM KIAA1840-E25-F | 1.5 |
| 5uM KIAA1840-E25-R | 1.5 |
| 10×buffer | 2.5 |
| r-Taq | 0.2 |
| template | 2 |

5. Procedure：

94℃5min→94℃30S→59℃30S→72℃45S→72℃10min→4℃

×35

**KIAA1840-E30A**

1.KIAA1840-E30A-F：tga ggt ggg agg atc tct tg

2.KIAA1840-E30A-R：gat gtg ttc aga gca gcc aa

3.length：622bp

4.system：

|  | 1× |
| --- | --- |
| ddH_2_O | 4.8 |
| 2mM dNTP | 2.5 |
| 5uM - KIAA1840-E30A-F | 1.5 |
| 5uM - KIAA1840-E30A-R | 1.5 |
| 2×GC buffer Ⅰ | 12.5 |
| LA-Taq | 0.2 |
| template | 2 |

5. Procedure：

94℃5min→94℃30S→66℃30S→72℃45S→94℃30S→51℃30S→72℃45S→72℃10min→4℃

×15（-1℃/cycle）

×20

**KIAA1840-E30B**

1.KIAA1840-E30B-F：taa gct gga gga gct gga ga

2.KIAA1840-E30B-R：ttg ttg tcc cct taa ctt gg

3.length：603bp

4.system：

|  | 1× |
| --- | --- |
| ddH_2_O | 4.8 |
| 2mM dNTP | 2.5 |
| 5uM - KIAA1840-E30B-F | 1.5 |
| 5uM - KIAA1840-E30B-R | 1.5 |
| 2×GC buffer Ⅰ | 12.5 |
| LA-Taq | 0.2 |
| template | 2 |

5. Procedure：

94℃5min→94℃30S→66℃30S→72℃45S→94℃30S→51℃30S→72℃45S→72℃10min→4℃

×20

×15（-1℃/cycle）

**KIAA1840-E31**

1.KIAA1840-E31-F：ttt gaa gta tcc cag ggt gg

2.KIAA1840-E31-R：cca cca ttc ccc aag ata a

3.length：510bp

4.system：

|  | 1× |
| --- | --- |
| ddH_2_O | 14.8 |
| 2mM dNTP | 2.5 |
| 5uM KIAA1840-E31-F | 1.5 |
| 5uM KIAA1840-E31-R | 1.5 |
| 10×buffer | 2.5 |
| r-Taq | 0.2 |
| template | 2 |

5. Procedure：

94℃5min→94℃30S→57℃30S→72℃45S→72℃10min→4℃

×35

**KIAA1840-E32**

1.KIAA1840-E32-F：tta cct gga ttt ggc tttg g

2.KIAA1840-E32-R：tgc aat cca gaa act tga gag a

3.length：517bp

4.system：

|  | 1× |
| --- | --- |
| ddH_2_O | 14.8 |
| 2mM dNTP | 2.5 |
| 5uM KIAA1840-E32-F | 1.5 |
| 5uM KIAA1840-E32-R | 1.5 |
| 10×buffer | 2.5 |
| r-Taq | 0.2 |
| template | 2 |

5. Procedure：

94℃5min→94℃30S→60℃30S→72℃45S→72℃10min→4℃

×35

**KIAA1840-E33**

1.KIAA1840-E33-F：caa tag gcc aag ggt tt caa

2.KIAA1840-E33-R：tat aac tcc tgc tgg agg gc

3.length：373bp

4.system：

|  | 1× |
| --- | --- |
| ddH_2_O | 14.8 |
| 2mM dNTP | 2.5 |
| 5uM KIAA1840-E33-F | 1.5 |
| 5uM KIAA1840-E33-R | 1.5 |
| 10×buffer | 2.5 |
| r-Taq | 0.2 |
| template | 2 |

5.procedure：

94℃5min→94℃30S→59℃30S→72℃45S→72℃10min→4℃

×35

**KIAA1840-E34**

1.KIAA1840-E34-F：atg ttg gca gga act cca tc

2.KIAA1840-E34-R：ctc ctt tgg agc aac ctc tg

3.length：373bp

4.system：

|  | 1× |
| --- | --- |
| ddH_2_O | 14.8 |
| 2mM dNTP | 2.5 |
| 5uM KIAA1840-E34-F | 1.5 |
| 5uM KIAA1840-E34-R | 1.5 |
| 10×buffer | 2.5 |
| r-Taq | 0.2 |
| template | 2 |

5.procedure：

94℃5min→94℃30S→59℃30S→72℃45S→72℃10min→4℃

×35

**KIAA1840-E35**

1.KIAA1840-E35-F：ggt agc ctg gaa att agc cc

2.KIAA1840-E35-R：tga acc aga atc tga agc ca

3.length：408bp

4.system：

|  | 1× |
| --- | --- |
| ddH_2_O | 14.8 |
| 2mM dNTP | 2.5 |
| 5uM KIAA1840-E35-F | 1.5 |
| 5uM KIAA1840-E35-R | 1.5 |
| 10×buffer | 2.5 |
| r-Taq | 0.2 |
| template | 2 |

5.procedure：

94℃5min→94℃30S→63℃30S→72℃45S→72℃10min→4℃

×35

**KIAA1840-E36**

1.KIAA1840-E36-F：GCGGGTGTATTTTTAAGGTTTC

2.KIAA1840-E36-R：AGCAGCACTGTTCTGGTAGTGT

3.length：449bp

4.system：

|  | 1× |
| --- | --- |
| ddH_2_O | 4.8 |
| 2mM dNTP | 2.5 |
| 5uM KIAA1840-E36-cyy-F | 1.5 |
| 5uM KIAA1840-E36-cyy-R | 1.5 |
| 2×GCbufferI | 12.5 |
| LA-Taq | 0.2 |
| template | 2 |

5.procedure：

94℃5min→94℃30S→62℃30S→72℃45S→72℃10min→4℃

×35

**KIAA1840-E37**

1.KIAA1840-E37- F：gca tta gaa ggg gca ctg aa

2.KIAA1840-E37- R：ctc aca acg gta ttc acc cc

3.length：443bp

4.system：

|  | 1× |
| --- | --- |
| ddH_2_O | 14.8 |
| 2mM dNTP | 2.5 |
| 5uM KIAA1840-E37-F | 1.5 |
| 5uM KIAA1840-E37-R | 1.5 |
| 10×buffer | 2.5 |
| r-Taq | 0.2 |
| template | 2 |

5.procedure：

94℃5min→94℃30S→64℃30S72℃45S→72℃10min→4℃

×35

**KIAA1840-E38**

1.KIAA1840-E38-F：ttt tgt cct tgg gct ctt tc

2.KIAA1840-E38-R：cct ggt tct gtc act agc cc

3.length：405bp

4.system：

|  | 1× |
| --- | --- |
| ddH_2_O | 14.8 |
| 2mM dNTP | 2.5 |
| 5uM KIAA1840-E38-F | 1.5 |
| 5uM KIAA1840-E38-R | 1.5 |
| 10×buffer | 2.5 |
| r-Taq | 0.2 |
| template | 2 |

5.procedure：

94℃5min→94℃30S→63℃30S72℃45S→72℃10min→4℃

×35

**KIAA1840-E39**

1.KIAA1840-E39-F：aag ggt tta aga taa ttt ggg ga

2.KIAA1840-E39-R：gga ttc ttg ata ctg ctt tgc c

3.length：503bp

4.system：

|  | 1× |
| --- | --- |
| ddH_2_O | 14.8 |
| 2mM dNTP | 2.5 |
| 5uM KIAA1840-E39-F | 1.5 |
| 5uM KIAA1840-E39-R | 1.5 |
| 10×buffer | 2.5 |
| r-Taq | 0.2 |
| template | 2 |

5.procedure：

94℃5min→94℃30S→59℃30S℃72℃45S→72℃10min→4℃

×35

**KIAA1840-E40A**

1.KIAA1840-E40A-F：aat tag cca ggg tgg tga ca

2.KIAA1840-E40A-R：ccc aca aag gac tga tat gg

3.length：516bp

4.system：

|  | 1× |
| --- | --- |
| ddH_2_O | 4.8 |
| 2mM dNTP | 2.5 |
| 5uM - KIAA1840-E40A-F | 1.5 |
| 5uM - KIAA1840-E40A-R | 1.5 |
| 2×GC buffer Ⅰ | 12.5 |
| LA-Taq | 0.2 |
| template | 2 |

5.procedure：

94℃5min→94℃30S→65℃30S→72℃45S→94℃30S→50℃30S→72℃45S→72℃10min→4℃

×20

×15（-1℃/cycle）

**KIAA1840-E40B**

1.KIAA1840-E40B-F：aag gac cct cag aca ggt tg

2.KIAA1840-E40B-R：tcc ttt aag gca gac aag gg

3.length：526bp

4.system：

|  | 1× |
| --- | --- |
| ddH_2_O | 14.8 |
| 2mM dNTP | 2.5 |
| 5uM KIAA1840-E40B-F | 1.5 |
| 5uM KIAA1840-E40B-R | 1.5 |
| 10×buffer | 2.5 |
| r-Taq | 0.2 |
| template | 2 |

5.procedure：

94℃5min→94℃30S→63℃30S72℃45S→72℃10min→4℃

×35
