# Supplementary material for: A case report of SPG11 mutations in a Chinese ARHSP-TCC family
Source: BMC Neurol. 2016 Jun 3;16:87. doi: 10.1186/s12883-016-0604-5 (PMC4891852; doi:10.1186/s12883-016-0604-5)
Supplement: Additional file 2: — Splice Site Prediction by Neural Network. (PPTX 77 kb) [file 12883_2016_604_MOESM2_ESM.pptx]

## Slide 1
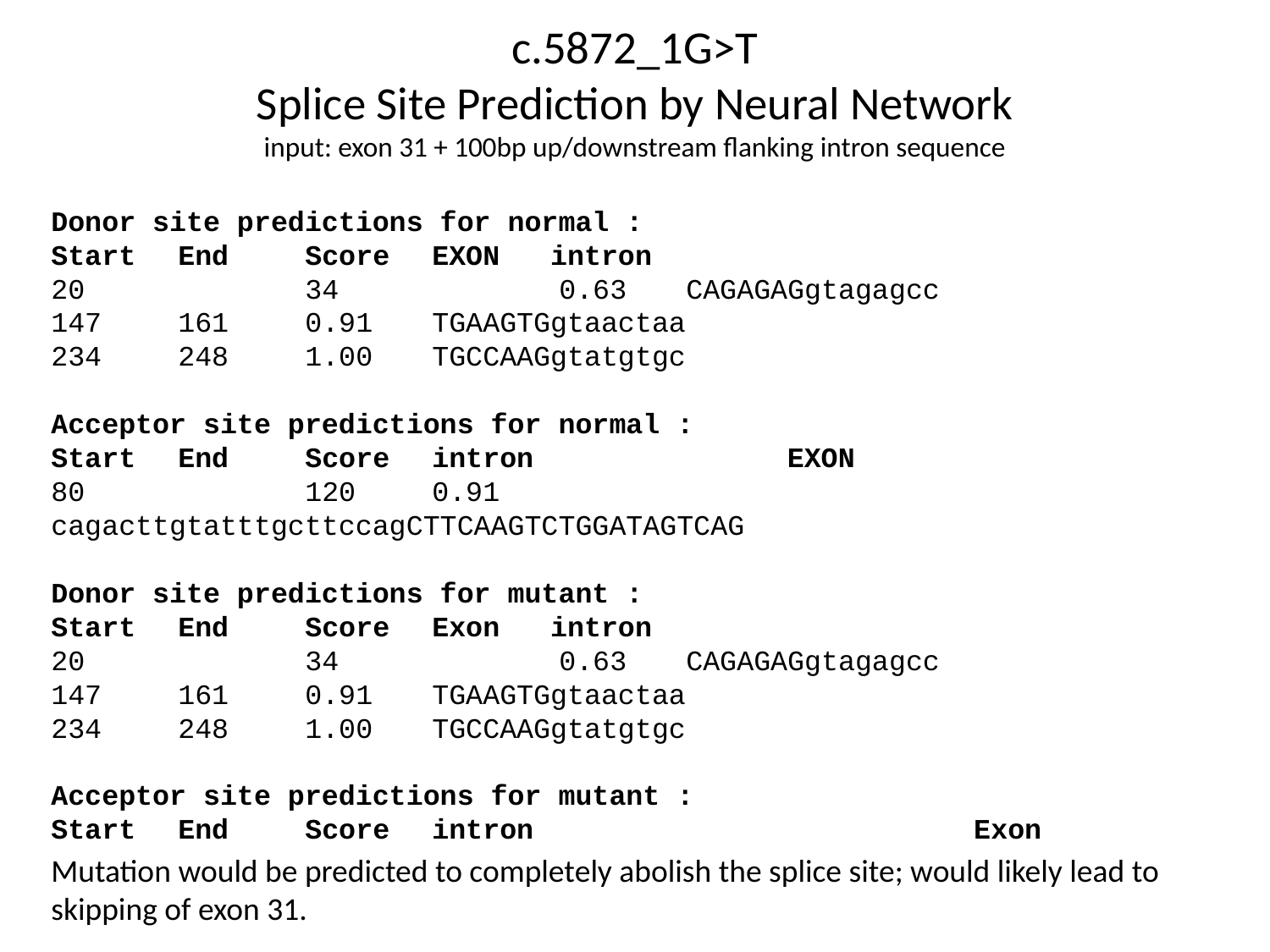

# c.5872_1G>T Splice Site Prediction by Neural Network input: exon 31 + 100bp up/downstream flanking intron sequence
Donor site predictions for normal :
Start 	End 	Score 	EXON intron
20 		34 		0.63 	CAGAGAGgtagagcc
147 	161 	0.91 	TGAAGTGgtaactaa
234 	248 	1.00 	TGCCAAGgtatgtgc
Acceptor site predictions for normal :
Start 	End 	Score 	intron EXON
80 		120 	0.91 	cagacttgtatttgcttccagCTTCAAGTCTGGATAGTCAG
Donor site predictions for mutant :
Start 	End 	Score 	Exon intron
20 		34 		0.63 	CAGAGAGgtagagcc
147 	161 	0.91 	TGAAGTGgtaactaa
234 	248 	1.00 	TGCCAAGgtatgtgc
Acceptor site predictions for mutant :
Start 	End 	Score 	intron 				 Exon
Mutation would be predicted to completely abolish the splice site; would likely lead to skipping of exon 31.

## Slide 2
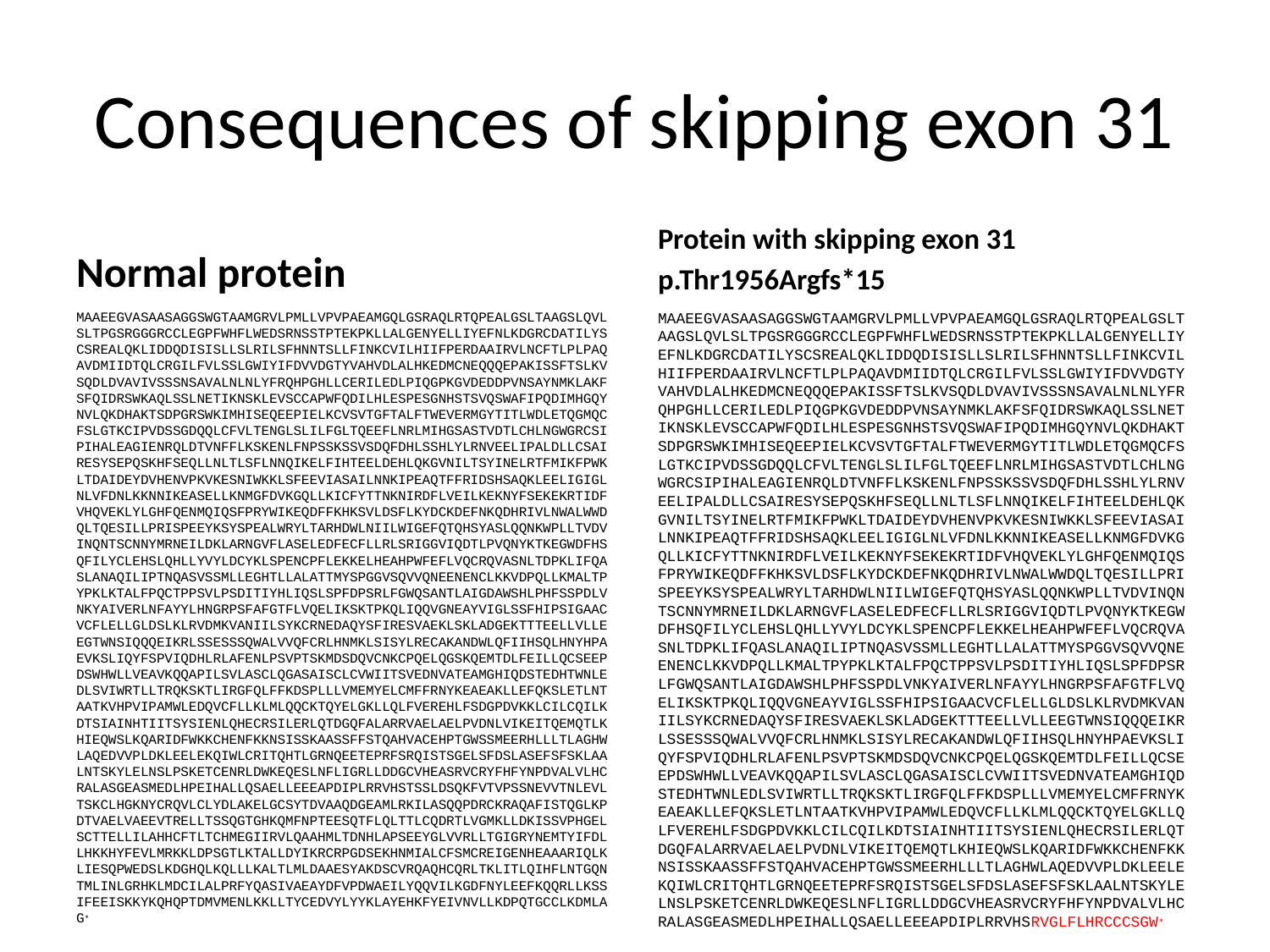

# Consequences of skipping exon 31
Normal protein
Protein with skipping exon 31
p.Thr1956Argfs*15
MAAEEGVASAASAGGSWGTAAMGRVLPMLLVPVPAEAMGQLGSRAQLRTQPEALGSLTAAGSLQVLSLTPGSRGGGRCCLEGPFWHFLWEDSRNSSTPTEKPKLLALGENYELLIYEFNLKDGRCDATILYSCSREALQKLIDDQDISISLLSLRILSFHNNTSLLFINKCVILHIIFPERDAAIRVLNCFTLPLPAQAVDMIIDTQLCRGILFVLSSLGWIYIFDVVDGTYVAHVDLALHKEDMCNEQQQEPAKISSFTSLKVSQDLDVAVIVSSSNSAVALNLNLYFRQHPGHLLCERILEDLPIQGPKGVDEDDPVNSAYNMKLAKFSFQIDRSWKAQLSSLNETIKNSKLEVSCCAPWFQDILHLESPESGNHSTSVQSWAFIPQDIMHGQYNVLQKDHAKTSDPGRSWKIMHISEQEEPIELKCVSVTGFTALFTWEVERMGYTITLWDLETQGMQCFSLGTKCIPVDSSGDQQLCFVLTENGLSLILFGLTQEEFLNRLMIHGSASTVDTLCHLNGWGRCSIPIHALEAGIENRQLDTVNFFLKSKENLFNPSSKSSVSDQFDHLSSHLYLRNVEELIPALDLLCSAIRESYSEPQSKHFSEQLLNLTLSFLNNQIKELFIHTEELDEHLQKGVNILTSYINELRTFMIKFPWKLTDAIDEYDVHENVPKVKESNIWKKLSFEEVIASAILNNKIPEAQTFFRIDSHSAQKLEELIGIGLNLVFDNLKKNNIKEASELLKNMGFDVKGQLLKICFYTTNKNIRDFLVEILKEKNYFSEKEKRTIDFVHQVEKLYLGHFQENMQIQSFPRYWIKEQDFFKHKSVLDSFLKYDCKDEFNKQDHRIVLNWALWWDQLTQESILLPRISPEEYKSYSPEALWRYLTARHDWLNIILWIGEFQTQHSYASLQQNKWPLLTVDVINQNTSCNNYMRNEILDKLARNGVFLASELEDFECFLLRLSRIGGVIQDTLPVQNYKTKEGWDFHSQFILYCLEHSLQHLLYVYLDCYKLSPENCPFLEKKELHEAHPWFEFLVQCRQVASNLTDPKLIFQASLANAQILIPTNQASVSSMLLEGHTLLALATTMYSPGGVSQVVQNEENENCLKKVDPQLLKMALTPYPKLKTALFPQCTPPSVLPSDITIYHLIQSLSPFDPSRLFGWQSANTLAIGDAWSHLPHFSSPDLVNKYAIVERLNFAYYLHNGRPSFAFGTFLVQELIKSKTPKQLIQQVGNEAYVIGLSSFHIPSIGAACVCFLELLGLDSLKLRVDMKVANIILSYKCRNEDAQYSFIRESVAEKLSKLADGEKTTTEELLVLLEEGTWNSIQQQEIKRLSSESSSQWALVVQFCRLHNMKLSISYLRECAKANDWLQFIIHSQLHNYHPAEVKSLIQYFSPVIQDHLRLAFENLPSVPTSKMDSDQVCNKCPQELQGSKQEMTDLFEILLQCSEEPDSWHWLLVEAVKQQAPILSVLASCLQGASAISCLCVWIITSVEDNVATEAMGHIQDSTEDHTWNLEDLSVIWRTLLTRQKSKTLIRGFQLFFKDSPLLLVMEMYELCMFFRNYKEAEAKLLEFQKSLETLNTAATKVHPVIPAMWLEDQVCFLLKLMLQQCKTQYELGKLLQLFVEREHLFSDGPDVKKLCILCQILKDTSIAINHTIITSYSIENLQHECRSILERLQTDGQFALARRVAELAELPVDNLVIKEITQEMQTLKHIEQWSLKQARIDFWKKCHENFKKNSISSKAASSFFSTQAHVACEHPTGWSSMEERHLLLTLAGHWLAQEDVVPLDKLEELEKQIWLCRITQHTLGRNQEETEPRFSRQISTSGELSFDSLASEFSFSKLAALNTSKYLELNSLPSKETCENRLDWKEQESLNFLIGRLLDDGCVHEASRVCRYFHFYNPDVALVLHCRALASGEASMEDLHPEIHALLQSAELLEEEAPDIPLRRVHSTSSLDSQKFVTVPSSNEVVTNLEVLTSKCLHGKNYCRQVLCLYDLAKELGCSYTDVAAQDGEAMLRKILASQQPDRCKRAQAFISTQGLKPDTVAELVAEEVTRELLTSSQGTGHKQMFNPTEESQTFLQLTTLCQDRTLVGMKLLDKISSVPHGELSCTTELLILAHHCFTLTCHMEGIIRVLQAAHMLTDNHLAPSEEYGLVVRLLTGIGRYNEMTYIFDLLHKKHYFEVLMRKKLDPSGTLKTALLDYIKRCRPGDSEKHNMIALCFSMCREIGENHEAAARIQLKLIESQPWEDSLKDGHQLKQLLLKALTLMLDAAESYAKDSCVRQAQHCQRLTKLITLQIHFLNTGQNTMLINLGRHKLMDCILALPRFYQASIVAEAYDFVPDWAEILYQQVILKGDFNYLEEFKQQRLLKSSIFEEISKKYKQHQPTDMVMENLKKLLTYCEDVYLYYKLAYEHKFYEIVNVLLKDPQTGCCLKDMLAG*
MAAEEGVASAASAGGSWGTAAMGRVLPMLLVPVPAEAMGQLGSRAQLRTQPEALGSLTAAGSLQVLSLTPGSRGGGRCCLEGPFWHFLWEDSRNSSTPTEKPKLLALGENYELLIYEFNLKDGRCDATILYSCSREALQKLIDDQDISISLLSLRILSFHNNTSLLFINKCVILHIIFPERDAAIRVLNCFTLPLPAQAVDMIIDTQLCRGILFVLSSLGWIYIFDVVDGTYVAHVDLALHKEDMCNEQQQEPAKISSFTSLKVSQDLDVAVIVSSSNSAVALNLNLYFRQHPGHLLCERILEDLPIQGPKGVDEDDPVNSAYNMKLAKFSFQIDRSWKAQLSSLNETIKNSKLEVSCCAPWFQDILHLESPESGNHSTSVQSWAFIPQDIMHGQYNVLQKDHAKTSDPGRSWKIMHISEQEEPIELKCVSVTGFTALFTWEVERMGYTITLWDLETQGMQCFSLGTKCIPVDSSGDQQLCFVLTENGLSLILFGLTQEEFLNRLMIHGSASTVDTLCHLNGWGRCSIPIHALEAGIENRQLDTVNFFLKSKENLFNPSSKSSVSDQFDHLSSHLYLRNVEELIPALDLLCSAIRESYSEPQSKHFSEQLLNLTLSFLNNQIKELFIHTEELDEHLQKGVNILTSYINELRTFMIKFPWKLTDAIDEYDVHENVPKVKESNIWKKLSFEEVIASAILNNKIPEAQTFFRIDSHSAQKLEELIGIGLNLVFDNLKKNNIKEASELLKNMGFDVKGQLLKICFYTTNKNIRDFLVEILKEKNYFSEKEKRTIDFVHQVEKLYLGHFQENMQIQSFPRYWIKEQDFFKHKSVLDSFLKYDCKDEFNKQDHRIVLNWALWWDQLTQESILLPRISPEEYKSYSPEALWRYLTARHDWLNIILWIGEFQTQHSYASLQQNKWPLLTVDVINQNTSCNNYMRNEILDKLARNGVFLASELEDFECFLLRLSRIGGVIQDTLPVQNYKTKEGWDFHSQFILYCLEHSLQHLLYVYLDCYKLSPENCPFLEKKELHEAHPWFEFLVQCRQVASNLTDPKLIFQASLANAQILIPTNQASVSSMLLEGHTLLALATTMYSPGGVSQVVQNEENENCLKKVDPQLLKMALTPYPKLKTALFPQCTPPSVLPSDITIYHLIQSLSPFDPSRLFGWQSANTLAIGDAWSHLPHFSSPDLVNKYAIVERLNFAYYLHNGRPSFAFGTFLVQELIKSKTPKQLIQQVGNEAYVIGLSSFHIPSIGAACVCFLELLGLDSLKLRVDMKVANIILSYKCRNEDAQYSFIRESVAEKLSKLADGEKTTTEELLVLLEEGTWNSIQQQEIKRLSSESSSQWALVVQFCRLHNMKLSISYLRECAKANDWLQFIIHSQLHNYHPAEVKSLIQYFSPVIQDHLRLAFENLPSVPTSKMDSDQVCNKCPQELQGSKQEMTDLFEILLQCSEEPDSWHWLLVEAVKQQAPILSVLASCLQGASAISCLCVWIITSVEDNVATEAMGHIQDSTEDHTWNLEDLSVIWRTLLTRQKSKTLIRGFQLFFKDSPLLLVMEMYELCMFFRNYKEAEAKLLEFQKSLETLNTAATKVHPVIPAMWLEDQVCFLLKLMLQQCKTQYELGKLLQLFVEREHLFSDGPDVKKLCILCQILKDTSIAINHTIITSYSIENLQHECRSILERLQTDGQFALARRVAELAELPVDNLVIKEITQEMQTLKHIEQWSLKQARIDFWKKCHENFKKNSISSKAASSFFSTQAHVACEHPTGWSSMEERHLLLTLAGHWLAQEDVVPLDKLEELEKQIWLCRITQHTLGRNQEETEPRFSRQISTSGELSFDSLASEFSFSKLAALNTSKYLELNSLPSKETCENRLDWKEQESLNFLIGRLLDDGCVHEASRVCRYFHFYNPDVALVLHCRALASGEASMEDLHPEIHALLQSAELLEEEAPDIPLRRVHSRVGLFLHRCCCSGW*

## Slide 3
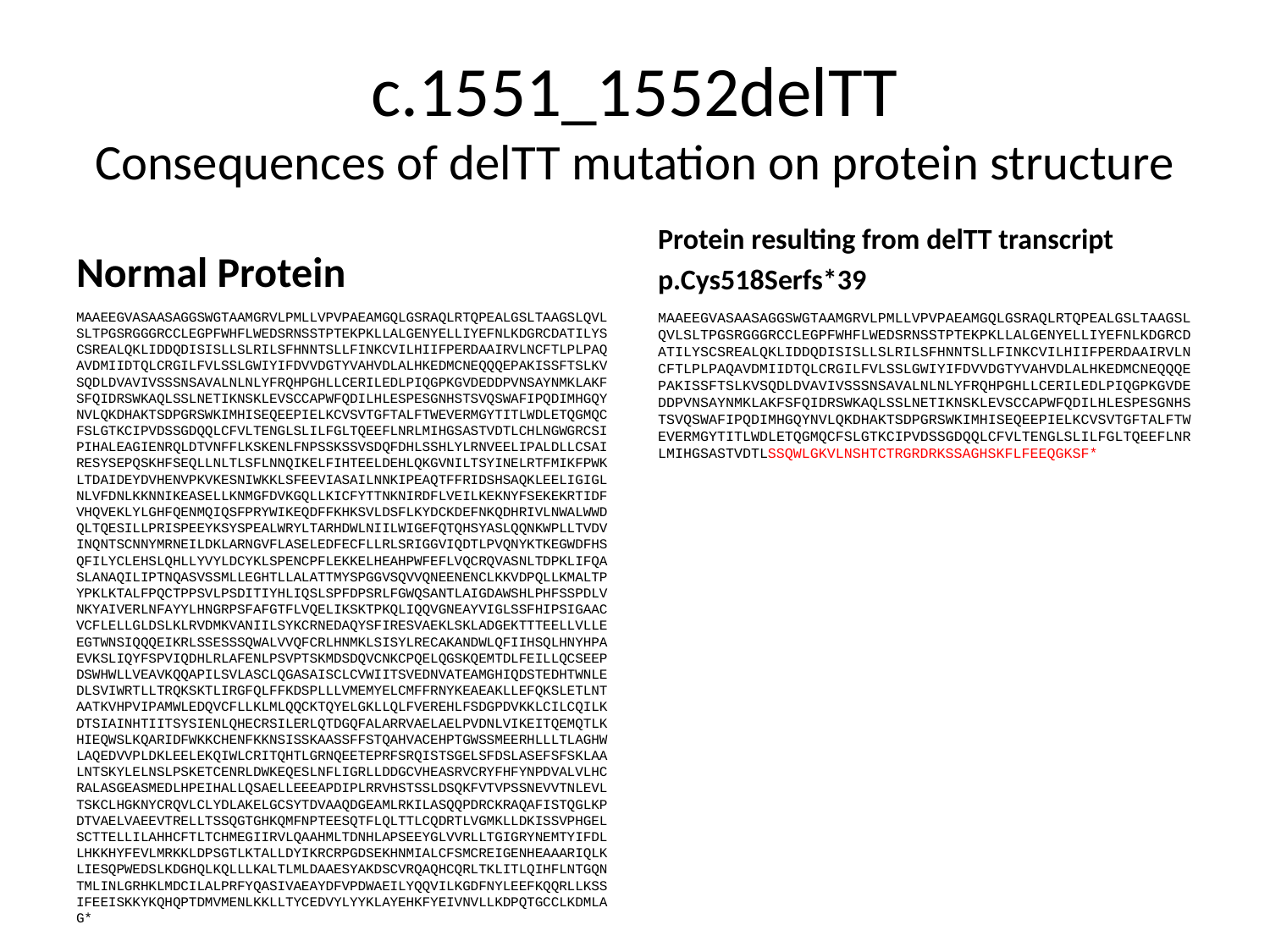

# c.1551_1552delTT Consequences of delTT mutation on protein structure
Normal Protein
Protein resulting from delTT transcript
p.Cys518Serfs*39
MAAEEGVASAASAGGSWGTAAMGRVLPMLLVPVPAEAMGQLGSRAQLRTQPEALGSLTAAGSLQVLSLTPGSRGGGRCCLEGPFWHFLWEDSRNSSTPTEKPKLLALGENYELLIYEFNLKDGRCDATILYSCSREALQKLIDDQDISISLLSLRILSFHNNTSLLFINKCVILHIIFPERDAAIRVLNCFTLPLPAQAVDMIIDTQLCRGILFVLSSLGWIYIFDVVDGTYVAHVDLALHKEDMCNEQQQEPAKISSFTSLKVSQDLDVAVIVSSSNSAVALNLNLYFRQHPGHLLCERILEDLPIQGPKGVDEDDPVNSAYNMKLAKFSFQIDRSWKAQLSSLNETIKNSKLEVSCCAPWFQDILHLESPESGNHSTSVQSWAFIPQDIMHGQYNVLQKDHAKTSDPGRSWKIMHISEQEEPIELKCVSVTGFTALFTWEVERMGYTITLWDLETQGMQCFSLGTKCIPVDSSGDQQLCFVLTENGLSLILFGLTQEEFLNRLMIHGSASTVDTLCHLNGWGRCSIPIHALEAGIENRQLDTVNFFLKSKENLFNPSSKSSVSDQFDHLSSHLYLRNVEELIPALDLLCSAIRESYSEPQSKHFSEQLLNLTLSFLNNQIKELFIHTEELDEHLQKGVNILTSYINELRTFMIKFPWKLTDAIDEYDVHENVPKVKESNIWKKLSFEEVIASAILNNKIPEAQTFFRIDSHSAQKLEELIGIGLNLVFDNLKKNNIKEASELLKNMGFDVKGQLLKICFYTTNKNIRDFLVEILKEKNYFSEKEKRTIDFVHQVEKLYLGHFQENMQIQSFPRYWIKEQDFFKHKSVLDSFLKYDCKDEFNKQDHRIVLNWALWWDQLTQESILLPRISPEEYKSYSPEALWRYLTARHDWLNIILWIGEFQTQHSYASLQQNKWPLLTVDVINQNTSCNNYMRNEILDKLARNGVFLASELEDFECFLLRLSRIGGVIQDTLPVQNYKTKEGWDFHSQFILYCLEHSLQHLLYVYLDCYKLSPENCPFLEKKELHEAHPWFEFLVQCRQVASNLTDPKLIFQASLANAQILIPTNQASVSSMLLEGHTLLALATTMYSPGGVSQVVQNEENENCLKKVDPQLLKMALTPYPKLKTALFPQCTPPSVLPSDITIYHLIQSLSPFDPSRLFGWQSANTLAIGDAWSHLPHFSSPDLVNKYAIVERLNFAYYLHNGRPSFAFGTFLVQELIKSKTPKQLIQQVGNEAYVIGLSSFHIPSIGAACVCFLELLGLDSLKLRVDMKVANIILSYKCRNEDAQYSFIRESVAEKLSKLADGEKTTTEELLVLLEEGTWNSIQQQEIKRLSSESSSQWALVVQFCRLHNMKLSISYLRECAKANDWLQFIIHSQLHNYHPAEVKSLIQYFSPVIQDHLRLAFENLPSVPTSKMDSDQVCNKCPQELQGSKQEMTDLFEILLQCSEEPDSWHWLLVEAVKQQAPILSVLASCLQGASAISCLCVWIITSVEDNVATEAMGHIQDSTEDHTWNLEDLSVIWRTLLTRQKSKTLIRGFQLFFKDSPLLLVMEMYELCMFFRNYKEAEAKLLEFQKSLETLNTAATKVHPVIPAMWLEDQVCFLLKLMLQQCKTQYELGKLLQLFVEREHLFSDGPDVKKLCILCQILKDTSIAINHTIITSYSIENLQHECRSILERLQTDGQFALARRVAELAELPVDNLVIKEITQEMQTLKHIEQWSLKQARIDFWKKCHENFKKNSISSKAASSFFSTQAHVACEHPTGWSSMEERHLLLTLAGHWLAQEDVVPLDKLEELEKQIWLCRITQHTLGRNQEETEPRFSRQISTSGELSFDSLASEFSFSKLAALNTSKYLELNSLPSKETCENRLDWKEQESLNFLIGRLLDDGCVHEASRVCRYFHFYNPDVALVLHCRALASGEASMEDLHPEIHALLQSAELLEEEAPDIPLRRVHSTSSLDSQKFVTVPSSNEVVTNLEVLTSKCLHGKNYCRQVLCLYDLAKELGCSYTDVAAQDGEAMLRKILASQQPDRCKRAQAFISTQGLKPDTVAELVAEEVTRELLTSSQGTGHKQMFNPTEESQTFLQLTTLCQDRTLVGMKLLDKISSVPHGELSCTTELLILAHHCFTLTCHMEGIIRVLQAAHMLTDNHLAPSEEYGLVVRLLTGIGRYNEMTYIFDLLHKKHYFEVLMRKKLDPSGTLKTALLDYIKRCRPGDSEKHNMIALCFSMCREIGENHEAAARIQLKLIESQPWEDSLKDGHQLKQLLLKALTLMLDAAESYAKDSCVRQAQHCQRLTKLITLQIHFLNTGQNTMLINLGRHKLMDCILALPRFYQASIVAEAYDFVPDWAEILYQQVILKGDFNYLEEFKQQRLLKSSIFEEISKKYKQHQPTDMVMENLKKLLTYCEDVYLYYKLAYEHKFYEIVNVLLKDPQTGCCLKDMLAG*
MAAEEGVASAASAGGSWGTAAMGRVLPMLLVPVPAEAMGQLGSRAQLRTQPEALGSLTAAGSLQVLSLTPGSRGGGRCCLEGPFWHFLWEDSRNSSTPTEKPKLLALGENYELLIYEFNLKDGRCDATILYSCSREALQKLIDDQDISISLLSLRILSFHNNTSLLFINKCVILHIIFPERDAAIRVLNCFTLPLPAQAVDMIIDTQLCRGILFVLSSLGWIYIFDVVDGTYVAHVDLALHKEDMCNEQQQEPAKISSFTSLKVSQDLDVAVIVSSSNSAVALNLNLYFRQHPGHLLCERILEDLPIQGPKGVDEDDPVNSAYNMKLAKFSFQIDRSWKAQLSSLNETIKNSKLEVSCCAPWFQDILHLESPESGNHSTSVQSWAFIPQDIMHGQYNVLQKDHAKTSDPGRSWKIMHISEQEEPIELKCVSVTGFTALFTWEVERMGYTITLWDLETQGMQCFSLGTKCIPVDSSGDQQLCFVLTENGLSLILFGLTQEEFLNRLMIHGSASTVDTLSSQWLGKVLNSHTCTRGRDRKSSAGHSKFLFEEQGKSF*

## Slide 4
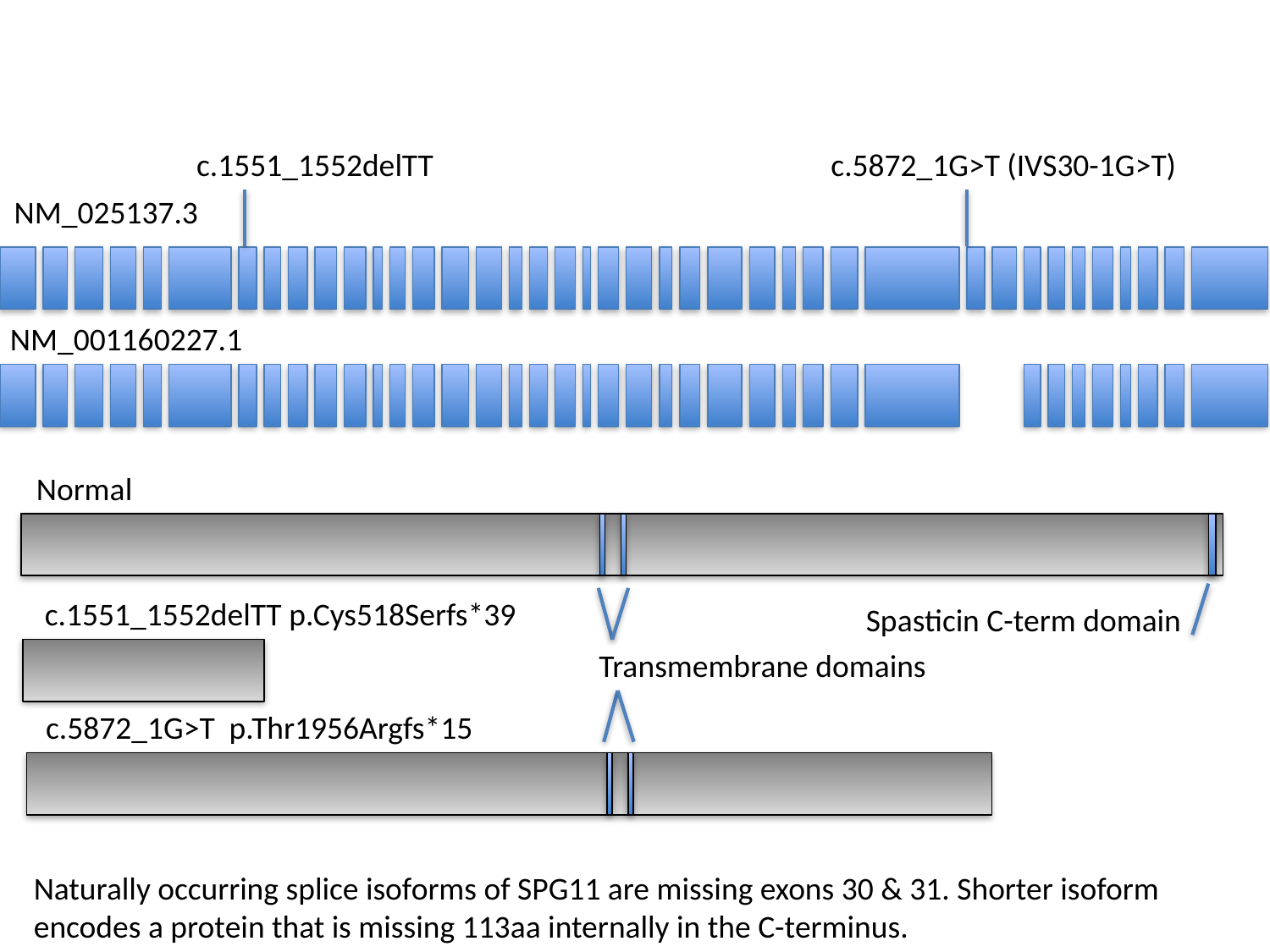

c.1551_1552delTT
c.5872_1G>T (IVS30-1G>T)
NM_025137.3
NM_001160227.1
Normal
c.1551_1552delTT p.Cys518Serfs*39
Spasticin C-term domain
Transmembrane domains
c.5872_1G>T p.Thr1956Argfs*15
Naturally occurring splice isoforms of SPG11 are missing exons 30 & 31. Shorter isoform encodes a protein that is missing 113aa internally in the C-terminus.
